# Editorial: The mechanistic and clinical principles of item-level scoring methods applied to the category fluency test and other tests of semantic memory

**DOI:** 10.3389/fpsyg.2023.1152574

**Published:** 2023-02-21

**Authors:** Matteo De Marco, Jet M. J. Vonk, Davide Quaranta

**Affiliations:** ^1^Department of Life Sciences, Brunel University London, London, United Kingdom; ^2^Department of Neurology, Memory and Aging Center, University of California, San Francisco, San Francisco, CA, United States; ^3^Department of Neuroscience, Catholic University of the Sacred Heart, Rome, Italy

**Keywords:** category fluency, item-based, semantic memory, Alzheimer's disease, preclinical

Implemented worldwide and based on quick and inexpensive methods of administration, standardized neuropsychological tests serve two main purposes. First, they are clinical instruments routinely used in diagnostic settings to characterize the cognitive profiles of single individuals. Second, they are methodological tools implemented in applied group/cohort-based research and in research aimed at elucidating mechanisms of normal and abnormal neurocognitive functioning. An important cognitive domain relevant to both these purposes is semantic memory, a form of memory for facts, concepts, and the meaning of words that has shown theory-informed and diagnostically-exploitable associations with the neuropathological and clinical features of Alzheimer's disease (AD) (Quaranta et al., 2019; Vonk et al., 2020).

Semantic memory can be measured with several tests, but one of the most well-known and popular tests for this domain is the category fluency test—naming as many items of a given category under time constraints. However, category fluency and other tests of semantic memory present a number of methodological aspects that may limit their clinical effectiveness. First, performance on these tests is in part supported by other non-semantic memory abilities that may be relatively spared by underlying pathological processes. Second, the efficiency of semantic memory processing depends on the integrity of encoding and retrieval abilities (as is the case for any memory sub-function), as well as on the wealth of knowledge that a person has accumulated throughout their life, and on the abilities of semantic control that are used to manipulate such knowledge. As a result, the construct validity of scoring procedures routinely used to quantify semantic memory test performance (e.g., total word count in category fluency) may be suboptimal in describing such a multidimensional concept.

Based on these methodological limitations, novel item-level methods can represent a valuable complementary approach. A shift of focus from global counts to item-based features offers the opportunity to explore novel and multifaceted scoring frameworks and can do so by relying on inexpensive post-processing methods without altering administration procedures. Additionally, these same principles can also apply to scoring other non-semantic memory tests to characterize the role played by semantic memory in supporting other functions, such as episodic memory. This article collection includes a selection of novel methods and approaches that share a common goal: optimizing the research-based and clinical use of verbal fluencies and other language-based tests.

In a concise position paper, Zemla reviewed the range of methodologies with which researchers have constructed models that capture the complexity of category fluency-informed semantic organization (with particular emphasis on mild cognitive impairment and AD). He particularly outlined the most substantial methodological issues that still need to be resolved, such as the lack of consensus over a set of procedural aspects.

De Marco and Venneri analyzed category fluency performance of 40 healthy adults and explored the neurofunctional correlates of semantic indices calculated on a series of ten item-based word properties. They associated item-level scores with the serial order of recall (i.e., the ordinal position of each word within its list) to define correlational scores indicative of the tendency to generate increasingly difficult words as the test progresses. They found that the ability to generate words that are increasingly less frequent, less dominant, and less interactable was associated with stronger expression of the default mode network in regions deputed to semantic control.

Paek administered a verb/action fluency to a sample of 17 cognitively healthy adults and 15 individuals with AD, and found that participants tended to produce words of higher emotional valence, and that valence was negatively correlated to memory performance.

Rofes et al. explored the performance of verbal fluency using supervised-learning regression methods in a group of 50 HIV-positive Argentinian participants to test the effect of item-based and inter-item category fluency descriptors on global category fluency scores. Participants' performance was analyzed using three distinct inferential models; across methods, cluster switching and the interaction term between cluster switching and word frequency showed to be the strongest predictors of quantitative performance.

A more complex and methodologically-rich approach was devised by Saranpää et al., who analyzed category fluency performance in 42 healthy controls, 24 individuals with amnestic mild cognitive impairment, and 18 individuals with early-stage AD. The authors reconstructed a semantic space using a neural-network model known as “word2vec” and a series of data-reduction methods. After defining eight animal sub-categories, the authors found that diagnostic status was predicted by word count and the number of returns to previously-covered sub-categories. This finding indicates that single items on category fluency are informative of how a category is explored thematically, and that AD could alter this exploration.

Bushnell et al. analyzed a sample of 640 individuals enrolled in the REGARDS study presenting with cognitive difficulties (as established by their performance on a screening task) and an equal number of matched controls. Using telephone-based verbal fluency recordings, they applied various methods to quantify clusters and switching during category and letter fluency. In one of their inferential models based on logistic regression discriminating between stable controls and progressive decliners, they found that the inclusion of speed scores when transitioning between connected and non-connected words offered a better prediction than the sole use of word count, perseverations, and intrusions.

Lastly, Mueller et al. applied item-level analyses beyond semantic memory tests by retrospectively analyzing a cohort of over 1,100 cognitively unimpaired participants to investigate the difficulty of items on the Logical Memory Test. They found that delayed performance, compared to the immediate recall, was based on the retrieval of more difficult items. They also reported that performance of individual items at discriminating between high and low performers was better in the delayed phase of recall, hinting at the possibility that semantic complexity might contribute to memorability of episodic details. Along similar lines, the discrimination index of several items was higher among amyloid-positive participants than amyloid-negative participants, suggesting that item-based memorability could be vulnerable to the pathological processes of AD.

Overall, these articles provide an overview of the heterogeneity of the approaches with which item-based properties of semantic memory and other memory scores can be operationalized and used in support of the traditional scoring methods. These novel methods hold promise at becoming valid methodological options that may be sensitive to the pathological alterations due to AD and other forms of cognitive impairment.

## Author contributions

MDM, JMJV, and DQ equally contributed to the writing up, editing, and revision of the manuscript. All authors contributed to the article and approved the submitted version.

## References

[B1] Quaranta D. Piccininni C. Caprara A. Malandrino A. Gainotti G. Marra C. (2019). Semantic relations in a categorical verbal fluency test: an exploratory investigation in mild cognitive impairment. Front. Psychol. 10, 2797. 10.3389/fpsyg.2019.02797 31920840 PMC6927990

[B2] Vonk J. M. J. Bouteloup V. Mangin J. F. Dubois B. Blanc F. Gabelle A.. (2020). Semantic loss marks early Alzheimer's disease-related neurodegeneration in older adults without dementia. Alzheimers Dement DADM. 12, e12066. 10.1002/dad2.12066 32775598 PMC7403823

